# Development and Characterization of Fe_3_O_4_@Carbon Nanoparticles and Their Biological Screening Related to Oral Administration

**DOI:** 10.3390/ma14133556

**Published:** 2021-06-25

**Authors:** Daniel Pop, Roxana Buzatu, Elena-Alina Moacă, Claudia Geanina Watz, Simona Cîntă Pînzaru, Lucian Barbu Tudoran, Fran Nekvapil, Ștefana Avram, Cristina Adriana Dehelean, Marius Octavian Crețu, Mirela Nicolov, Camelia Szuhanek, Anca Jivănescu

**Affiliations:** 1 Department of Prosthodontics, Faculty of Dental Medicine, “Victor Babes” University of Medicine and Pharmacy, Revolutiei Ave. 1989, No. 9, RO-300580 Timișoara, Romania; pop.daniel@umft.ro (D.P.); jivanescu.anca@umft.ro (A.J.); 2 TADERP Reseach Center—Advanced and Digital Techniques for Endodontic, Restorative and Prosthetic Treatment, “Victor Babeș” University of Medicine and Pharmacy, Revolutiei Ave. 1989, No. 9, RO-300041 Timişoara, Romania; 3 Department of Dental Aesthetics, Faculty of Dental Medicine, “Victor Babeș” University of Medicine and Pharmacy, Revolutiei Ave. 1989, No. 9, RO-300041 Timişoara, Romania; drbuzaturoxana@gmail.com; 4 Department of Toxicology and Drug Industry, Faculty of Pharmacy, “Victor Babeș” University of Medicine and Pharmacy Timisoara, 2nd Eftimie Murgu Square, RO-300041 Timisoara, Romania; cadehelean@umft.ro; 5 Research Centre for Pharmaco-Toxicological Evaluation, “Victor Babeș” University of Medicine and Pharmacy, 2nd Eftimie Murgu Square, RO-300041 Timișoara, Romania; stefana.avram@umft.ro; 6 Department of Pharmaceutical Physics, Faculty of Pharmacy, “Victor Babeș” University of Medicine and Pharmacy Timisoara, 2nd Eftimie Murgu Square, RO-300041 Timisoara, Romania; nicolovmirela@gmail.com; 7 Biomolecular Physics Department, Babes-Bolyai University, 1 Kogalniceanu Street, RO-400084 Cluj-Napoca, Romania; simona.pinzaru@ubbcluj.ro (S.C.P.); neki.fran@gmail.com (F.N.); 8 RDI Laboratory of Applied Raman Spectroscopy, RDI Institute of Applied Natural Sciences (IRDI-ANS), Babeş-Bolyai University, 42 Fântânele Street, RO-400293 Cluj-Napoca, Romania; 9 Electron Microscopy Laboratory “Prof. C. Craciun”, Faculty of Biology & Geology, “Babes-Bolyai” University, 5-7 Clinicilor Street, RO-400006 Cluj-Napoca, Romania; lucianbarbu@yahoo.com; 10 Electron Microscopy Integrated Laboratory, National Institute for R&D of Isotopic and Molecular Technologies, 67-103 Donat Street, RO-400293 Cluj-Napoca, Romania; 11 Department of Pharmacognosy, Faculty of Pharmacy, University of Medicine and Pharmacy “Victor Babeș” Timisoara, 2nd Eftimie Murgu Square, RO-300041 Timișoara, Romania; 12 Department of Surgery, Faculty of Medicine, “Victor Babes” University of Medicine and Pharmacy, 2nd Eftimie Murgu Square, RO-300041 Timisoara, Romania; tavicretu@yahoo.com; 13 Department of Orthodontics, Faculty of Dental Medicine, University of Medicine and Pharmacy “Victor Babes”, Timisoara, Revolutiei Ave. 1989, No. 9, RO-300041 Timisoara, Romania; cameliaszuhanek@umft.ro

**Keywords:** magnetite, combustion synthesis, Raman spectroscopy, magnetic measurements, HGF, cytotoxicity, HET-CAM assay

## Abstract

The current study presents the effect of naked Fe_3_O_4_@Carbon nanoparticles obtained by the combustion method on primary human gingival fibroblasts (HGFs) and primary gingival keratinocytes (PGKs)—relevant cell lines of buccal oral mucosa. In this regard, the objectives of this study were as follows: (i) development via combustion method and characterization of nanosized magnetite particles with carbon on their surface, (ii) biocompatibility assessment of the obtained magnetic nanoparticles on HGF and PGK cell lines and (iii) evaluation of possible irritative reaction of Fe_3_O_4_@Carbon nanoparticles on the highly vascularized chorioallantoic membrane of a chick embryo. Physicochemical properties of Fe_3_O_4_@Carbon nanoparticles were characterized in terms of phase composition, chemical structure, and polymorphic and molecular interactions of the chemical bonds within the nanomaterial, magnetic measurements, ultrastructure, morphology, and elemental composition. The X-ray diffraction analysis revealed the formation of magnetite as phase pure without any other secondary phases, and Raman spectroscopy exhibit that the pre-formed magnetic nanoparticles were covered with carbon film, resulting from the synthesis method employed. Scanning electron microscopy shown that nanoparticles obtained were uniformly distributed, with a nearly spherical shape with sizes at the nanometric level; iron, oxygen, and carbon were the only elements detected. While biological screening of Fe_3_O_4_@Carbon nanoparticles revealed no significant cytotoxic potential on the HGF and PGK cell lines, a slight sign of irritation was observed on a limited area on the chorioallantoic membrane of the chick embryo.

## 1. Introduction

Nowadays, nanotechnology is defined as a science of technology, which refers to the ability to engineer and utilizes materials as well as devices, at the nanometer level, with dimensions between 1–100 nm. On the other hand, nanotechnology includes the synthesis, characterization, and application of new materials with advanced properties and production of devices, as well as the study of material ultrastructure and morphology at the molecular and atomic scales [1,2,3,4]. Of great interest to most researchers dealing with nanotechnology science are nanoparticles due to the fact that the most of the research works reported in the literature have focused to interconnect the engineered science of these nanomaterials with medical problems. Nanoparticles are broadly used in all areas of medicine from drug development [5,6,7] to magnetic resonance imaging and hyperthermia for the treatment of cancer [8,9,10,11], targeted drug delivery [12,13], computer tomograf (CT) and optical imaging [14,15], cellular therapy [16,17], tissue repair [18], and gene manipulations [19,20].

In the last ten years, a huge interest in the development of new nanomaterials addressing dental applications has emerged. Due to the growing interest in designing new nanoscale materials for dentistry, a new field of research has been implemented, the so- called nano-dentistry [21,22,23,24,25]. Nano-dentistry refers to the development of new nanomaterials or devices which are planned to be in the first place in contact with buccal cavity flavoring the unpleasant breath, then in contact with the teeth, cleaning them, changing their appearance, thus preserving and improving dental health. Moreover, due to their unique and distinct biological properties, such as antimicrobial, antifungal, and antiviral properties, nanomaterials, more exactly nanoparticles, can be used in restorative dentistry, prosthetic dentistry, endodontics, implantology, biomineralization, oral cancers, and periodontology [26]. There are various dental nanomaterials suitable for all the above complications such as (i) organic (polymeric or lipid-based nanomaterials—e.g., polyethyleneglycol, solid-lipids, nanogels, dendrimers, chitosan); (ii) inorganic (metal, metal oxides, ceramics or semiconductors nanomaterials—e.g., zirconia, silica, titanium dioxide, hydroxyapatite, quantum dots), and (iii) nanocarbons, which comprise fullerenes, graphene, carbon nanotubes, carbon nanofiber or carbon black, which have been successfully used in therapeutic dentistry [26,27]. Carbon-based nanomaterials, particularly carbon nanotubes, have generated interest in the dentistry field because they lead to an improvement of strength of implants and composite materials, increase cell adhesion and proliferation, and provide protection against bacteria [28]. Graphene oxide, another nanocarbon material, is also a useful nanomaterial for regenerative dentistry and bone tissue engineering due to its physicomechanical properties; antimicrobial properties; low toxicity; ability to act as drug carriers toward specific organs and antitumor agents for oral cancer [29]. In addition to the many benefits that these nanomaterials possess, there are still growing concerns about the toxicological aspects of their use in dentistry. For example, the toxicity of silver nanoparticles is due to the activity of free silver ions released into the medium [30]; ZnO nanoparticles show cytotoxicity against human gingival fibroblasts, human embryonic lung fibroblasts, and gastric epithelial and neural stem cells [31,32]; gold nanoparticles seem to have prolonged retention within cells, and elimination difficulty by the kidney [33] associated with nanocomposites increases concerns regarding the inhalation of released nanoparticles during abrasive procedures [34].

Among the broad spectrum of nanoparticles, magnetic nanoparticles are promising nanomaterials in the field of biomedicine due to their excellent biocompatibility and biodegradability, non-toxicity, superparamagnetic properties, and solubility/stability in biological environments under physiological conditions [35,36]. In order to obtain these biomedical characteristics, it is necessary to choose a synthesis method which offers several advantages. To this end, from the myriad of synthesis methods that can be applied, the simplest and most economically advantageous and environmentally friendly proved to be the combustion method [37,38,39,40,41]. The interest in the combustion method is highlighted by the possibility of obtaining various nanomaterials with tailored properties, only by changing the synthesis conditions and controlling the parameters of the reaction [42], these representing the highest advantages of the combustion method. Another great advantage of the combustion method is the reaction time (several minutes) and the fact that the magnetic nanoparticles are obtained directly after combustion, without other subsequent process, therefore without energy consumption [43,44,45].

With applications in the dentistry field, research studies focus on the combination of magnetic nanoparticles with inorganic metals (such as silver) [46] used for endodontic disinfection or chitosan [47,48] for prednisolone delivery to the dental pulp, but none of these studies refers to the use of magnetic nanoparticles per se. Even so, the use of silver nanoparticles imposes significant drawbacks and must be avoided due to toxicity issues and the prolonged contact time required.

The purpose of the present study was as follows: (i) the manufacture of biocompatible magnetic nanoparticles via the combustion method; (ii) the evaluation of physicochemical features of the as-synthesized magnetic nanoparticles through the most used methods; (iii) assessment of the biological profile by employing an in vitro model based on relevant cells of buccal oral mucosa, using primary human gingival fibroblasts (HGFs) and primary gingival keratinocytes (PGKs) as the in vitro model and basic in vitro toxicological assays, such as cell viability quantification by means of the Alamar blue proliferation test and lactate dehydrogenase release method, along with cell morphology assessment at 24 h and 48 h post-stimulation, and iv) determination of the toxicological profile as a preclinical tool by assessment of the potential irritability of the Fe_3_O_4_@Carbon nanoparticles as well as their biocompatibility in an in vivo setting by employing the HET-CAM (hen’s egg chorioallantoic membrane) assay. The in vitro methods were selected based on previous research published by our group, where these types of techniques have already been employed successfully either for the biological assessment of Fe_3_O_4_ material [49,50,51] or for the evaluation of the biosecurity profile of different dental devices [52,53,54].

## 2. Materials and Methods

### 2.1. Chemicals and Reagents

The reagents used for the synthesis of Fe_3_O_4_ (magnetite) nanoparticles were iron nitrate nonahydrate—Fe(NO_3_)_3_·9H_2_O with a ≥96% purity, acquired from Carl Roth (Karlsruhe, Germany)—employed as oxidizing agent; citric acid monohydrate—C_6_H_8_O_7_·H_2_O, with a 99.5% purity, purchased from Silal Trading (Bucuresti, Romania)—employed as fuel; and distilled water—H_2_O_d_, acquired from Chemical Company SA (Iasi, Romania).

### 2.2. Cell Line and Cell Culture Protocol

The cell lines used in the current study are represented by primary human gingival fibroblasts (HGFs) and primary gingival keratinocytes (PGKs). The cells were obtained from the American Type Culture Collection (Manassas, VA, United States), code no. ATCC^®^ PCS-201-018^TM^ and ATCC^®^ PCS-200-014^TM^ (LGC Standards GmbH, Wesel, Germany) as frozen vials. The specific reagents used for the HGF cell line growth were supplied by ATCC, as follows: the cell culture medium—fibroblast basal medium, code no. ATCC^®^ PCS-201-030™, fibroblast growth kit-low serum, code no. ATCC^®^ PCS-201-041™, and 0.1% antibiotic mixture of penicillin–streptomycin–amphotericin B solution, code no. ATCC^®^ PCS-999-002™. The reagents used to culture the PGK cell line were the growth medium—dermal cell basal medium, code no. ATCC^®^ PCS-200-030^TM^, keratinocyte growth kit, code no. ATCC^®^ PCS-200-040 ^TM^, and 1% antibiotic mixture of 100 U/mL penicillin:100 µg/mL streptomycin. The cells were maintained in a Steri-Cycle i160 incubator (Thermo Fisher Scientific, Inc., Waltham, MA, USA) at 37 °C under a humidified atmosphere with 5% CO_2_. All in vitro techniques were conducted under sterile conditions using the MSC Advantage biosafety cabinet (12 model, Thermo Fisher Scientific, Inc., Waltham, MA, USA).

### 2.3. Synthesis of Magnetic Nanoparticles

The magnetite nanoparticles used in the current study were synthesized using an environmentally friendly method, simple and versatile, which involves low-cost raw materials, namely, the combustion method, according to the procedure thoroughly described by Ianos et al. [43]. The procedures involves the mixture of the iron nitrate nonahydrate—Fe(NO_3_)_3_·9H_2_O (0.09 mol) and citric acid monohydrate—C_6_H_8_O_7_·H_2_O (0.08 mol), in 25 mL distilled water, in order to yield 0.03 mol of Fe_3_O_4_. After complete dissolving of the reagents, the clear aqueous solution was rapidly heated using an in house built equipment, in the absence of air, consisting of a round bottom flask and a heating mantle, up to 400 °C for several minutes [43]. The combustion reaction was considered finished when the large amounts of gases that evolved during the combustion process stopped. Finally, a fluffy nanopowder, obtained after cooling to ambient temperature (24 °C), was hand ground and washed three times with hot distilled water (60–70 °C), magnetically decanted using a NdFeB block magnet (www.supermagnete.com (24.11.2020), Gottmadingen, Germany), and dried at 70 °C in an oven (POL-EKO Aparatura, Wodzisław Śląski, Poland). Throughout the cooling process of the fluffy nanopowder, the contact between the air and nanopowder was prevented, thus avoiding the oxidation of the magnetite nanoparticles.

### 2.4. Characterization of the Pre-Formed Magnetic Nanoparticles via the Combustion Method

The resulting magnetic nanoparticles were characterized by employing the following methods: X-ray diffraction, Brunauer, Emmett, Teller nitrogen gas adsorption technique, Raman Spectroscopy, Vibrating Sample Magnetometry, dynamic light scattering (DLS), and Scanning Electron Microscopy analysis.

#### 2.4.1. X-ray Diffraction (XRD)

XRD was used to establish the phase composition, purity, and degree of crystallinity, determined by a Rigaku Ultima IV diffractometer (Tokyo, Japan), with monocromated Cu-Kα radiation (1.5406 Å), operating at 40 kV and 40 mA. For peak assignment, we used only the PDF file specific for magnetite (1011032), from the International Centre for Diffraction Data Powder Diffraction File (ICDD PDF) 4+ 2019 data. By using the Debye-Sherrer’s equation, Equation (1), we calculated the crystallite size of the magnetic nanoparticles:

(1) $$D\;=\;\frac{0.9\;\cdot \;\lambda }{\beta \;\cdot \;\cos\theta }$$

where: D—the crystallite size (nm), λ—radiation wavelength (nm), β—the full width at half of the maximum in the 2θ scale (radians), θ—the Bragg angle.

#### 2.4.2. Brunauer, Emmett, Teller (BET) Nitrogen Gas Adsorption Technique

Using the BET nitrogen gas adsorption technique, we measured the specific surface area (S_BET_) of the magnetic nanoparticles, using a Micromeritics ASAP 2020 instrument (Micromeritics Instrument Corporation, Norcross, GA, USA). By applying Equation (2), we calculated the equivalent diameter:

(2) $$D_{BET}\;=\;\frac{6000}{\rho_{Fe_{3}O_{4}}\;\cdot \;S_{BET}}$$

where: $D_{BET}$—the grain size (nm), $\rho_{Fe_{3}O_{4}}$—the theoretical density of magnetite (5.2 g/cm^3^), $S_{BET}$—the BET surface area (m^2^/g).

#### 2.4.3. Raman Spectroscopy

In order to determine the vibrational modes of molecules contained in magnetic nanoparticles as well as other low-frequency modes of the magnetic sample, Raman Spectroscopy was used. Spectra with visible excitation were acquired using the Renishaw InVia Reflex confocal Raman microscope system (Wotton-under-Edge, UK). Samples of magnetic nanoparticles were deposited on a glass microscope slide and excited with a 532 nm laser line set to an output power of 10 mW, under a 20× objective (NA 0.35). Spectra were recorded on this device through the Wire 3.4 software, with a spectral resolution of 0.5 cm^−1^. As a supplementary analytical procedure, Fourier transform Raman spectra were recorded on a Bruker Equinox 55 spectrometer (Karlsruhe, Germany) with an integrated FRA 106/S Raman module. A Nd:YAG laser (Alphalas GmbH, Göttingen, Germany) emitting at 1064 nm with an output power of 350 mW was used for excitation, with the spectral resolution of 4 cm^−1^ recorded in a liquid nitrogen-cooled Ge detector.

#### 2.4.4. Scanning Electron Microscopy (SEM) Analysis with EDX Detectors

SEM analysis, a qualitative and semi-quantitative technique, was employed to establish the morphology of magnetic nanoparticles. The analysis was carried out on a Hitachi SU8230 cold field emission gun STEM (Chiyoda, Tokyo, Japan) scanning electron microscope with an EDX detector X-MaxN 80 from Oxford Instruments (Abingdon, UK). For better conductivity, in order to acquire high-resolution SEM imaging, the magnetic nanoparticles were sputter-coated with gold (6 nm) (Agar Automatic Sputtercoater, Stansted, UK). The parameters set for SEM analysis were HV (high vacuum) mode, 30 kV, acceleration voltage, secondary electron detectors (upper and lower), and two magnification orders, one to highlight the general aspect of magnetic nanoparticles and the other to show the nanomaterial surface topography. The identified chemical species were expressed both in atomic relative percent (At %) and weight relative percent (Wt %).

#### 2.4.5. DLS Technique

In order to establish the Fe_3_O_4_@Carbon particle size in suspension, we employed a precise measurement technique—DLS technique. The hydrodynamic diameter (Hd), poly dispersity index (PDI), and Zeta potential were measured by DLS using a Delsa Nano C particle analyzer from Beckman Coulter (Brea, CA, USA). By using the photon correlation spectroscopy, the particle size of Fe_3_O_4_@C was measured in aqueous suspension in a range from 0.6 nm to 7 µm. Zeta potential was measured by an electrophoretic light scattering technique using a flow cell. The measurement conditions were as follows: temperature 25 °C, distilled water (as a dispersant medium) with a refractive index of 1.3328, viscosity 0.8878 cP, and scattering intensity 9964 cps.

#### 2.4.6. Vibrating Sample Magnetometry

The obtained Fe_3_O_4_ nanoparticles were evaluated for their magnetic properties at room temperature (24 °C), using Vibrating Sample Magnetometry analysis (VSM), using a VSM 880 ADE/DMS magnetometer (DMS/ADE Technologies, Westwood, MA, USA). The external magnetic field applied ranged from 0 to 9·10^2^ kA/m.

### 2.5. Cell Morphology Evaluation

The morphological aspects of the HGF monolayer were monitored by taking pictures of the cells exposed to different concentrations (25, 50, 100, 125 µg/mL) of Fe_3_O_4_@Carbon nanoparticles. The pictures were taken using an Olympus IX73 inverted microscope with DP74 integrated camera (Olympus, Tokyo, Japan) at magnification 20×, immediately (0 h) after exposure of the cells to test samples and at 24 h and 48 h post-treatment.

### 2.6. Cell Viability Assessment by Means of the Alamar Blue Proliferation Test

In order to quantify the effect induced by Fe_3_O_4_@Carbon nanoparticles at concentrations of 25 µg/mL, 50 µg/mL, 100 µg/mL, 125 µg/mL on the viability of primary human gingival fibroblasts (HGFs) and primary human keratinocytes (PGKs), the Alamar blue colorimetric assay was applied. In brief, the protocol consisted of seeding the cells to an initial density of 10^4^ cells/well into 96-well plates. Further, the cells were incubated until a confluence of 80% was achieved, the old medium was removed, and the cells were treated with the test sample at concentrations of 25 µg/mL, 50 µg/mL, 100 µg/mL, 125 µg/mL for 24 h and 48 h. The control cells were treated only with cell culture medium and were maintained under the same conditions as the exposed cells.

The cell viability percentage was determined by reading the absorbance of the wells at least 3 h post-addition of the Alamar blue reagent. The absorbance was measured spectrophotometrically at two wavelengths of 570 nm and 600 nm, by using the microplate reader (xMarkTM Microplate, Bio-Rad Laboratories, Hercules, CA, USA). To quantify the cell viability percentage based on the determined absorbances, a previously published formula was used [54].

### 2.7. Cytotoxicity Assay via the LDH Release Method

To assess the cytotoxic activity induced by Fe_3_O_4_@Carbon nanoparticles at different concentrations (25 µg/mL, 50 µg/mL, 100 µg/mL, 125 µg/mL) on HGF and PGK monolayers, the lactate dehydrogenase release method was employed. The protocol used for this method is very similar to the one performed for the Alamar blue test. However, at the end of the stimulation period, 50 µL/well medium containing the LDH release was transferred in a new 96-well plate and then a 50 µL/well reaction mixture was added for 30 min and the plate was maintained at room temperature in a dark chamber. In the end, the reaction was stopped by addition 50 µL/well stop solution provided by the manufacturer (LDH assay kit, Code no 88954, Thermo Fisher Scientific, Boston, MA, USA).

To quantify the cytotoxic effect, the absorbance of each well was measured at 490 and 680 nm wavelengths by employing a microplate reader (xMarkTM Microplate, Bio-Rad Laboratories, Hercules, CA, USA).

### 2.8. HET-CAM Assay

The HET-CAM (hen’s egg chorioallantoic membrane) assay was performed in order to assess the potential irritability of the as-synthesized Fe_3_O_4_@Carbon nanoparticles as well as their biocompatibility in an in vivo setting, as a preclinical tool for the toxicologic profile of such a material. The HET—CAM test was performed, respecting the guideline of the Interagency Coordinating Committee on the Validation of Alternative Methods (ICCVAM) [55]. In brief, fertilized eggs were disinfected and incubated at 37 °C under controlled humidity. On the 10th day of development control (distilled water as a negative control, sodium lauryl sulphate (SLS) 0.5% in distilled water as a positive control) or test sample (125 µg/mL of Fe_3_O_4_@Carbon nanoparticles) was inoculated in volumes of 300 µL, and the reactions were monitored using CAM by means of stereomicroscopy (Discovery 8 Stereomicroscope, Zeiss, Stuttgart, Germany) for a duration of 300 s. Relevant images were captured (Axio CAM 105 color, Zeiss) before the application and after 5 min of contact with the sample. All images were processed using Zeiss ZEN software, Gimp 2.8, and ImageJ software.

The observation time of the produced reactions was 5 min (300 s), and the time at which the occurrence of a particular reaction was noted in seconds: hemorrhage—H (blood vessel bleeding), vascular lysis—L (disintegration of blood vessels), coagulation—C (intra- or extra- vascular protein denaturizing). With the registered data, an irritation score (IS) was calculated using the following equation:

(3) $$\mathrm{IS}\;=\;5\;\times \;\frac{301\;-\;\mathrm{Sec}\;H}{300}\;+\;7\;\times \;\frac{301\;-\;\mathrm{Sec}\;L}{300}\;+\;9\;\times \;\frac{301\;-\;\mathrm{Sec}\;C}{300}$$

where: H = hemorrhage; L = vessel lysis; C = coagulation; Hemorrhage time (Sec H) = onset of hemorrhage reactions on CAM (in seconds); Lysis time (Sec L) = onset of vessel lysis on CAM (in seconds); Coagulation time (Sec C) = onset of coagulation formation on CAM (in seconds). Means values were obtained. The IS values range on a scale between 0 and 21, as indicated by Luepke [56].

## 3. Results

### 3.1. XRD Analysis

**Figure 1:**
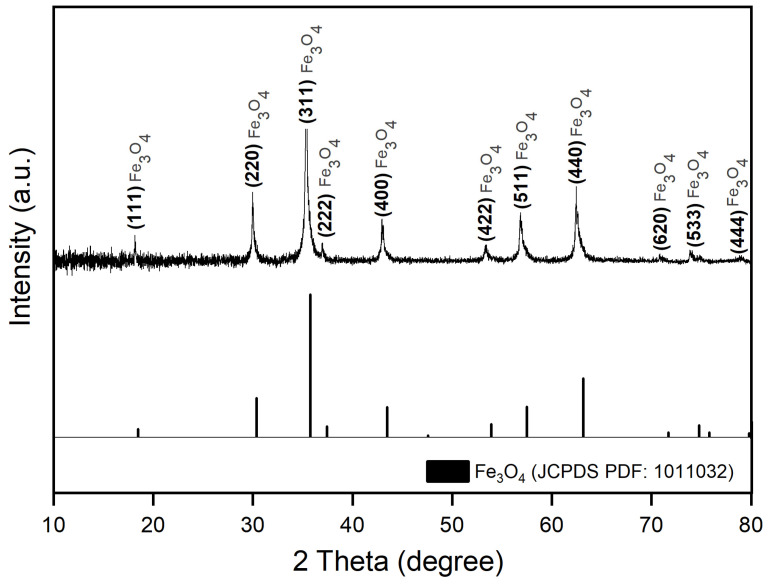
XRD pattern of magnetic nanoparticles, compared to the XRD patterns of magnetite, from the International Centre for Diffraction Data Powder Diffraction File (ICDD PDF) 4+ 2019 data.

The structure of magnetic nanoparticles was established by determining the phase composition and crystallite size estimation, using XRD analysis. The XRD pattern of the as-synthesized magnetic nanoparticles is shown in Figure 1, which exhibits the characteristic diffraction peaks for the formation of magnetite as a phase pure without any other secondary phases. The XRD pattern of the magnetite nanoparticles prepared via the combustion method exactly matched the parent pattern of magnetite (JCPDS file no. 1011032) from the International Centre for Diffraction Data Powder Diffraction File (ICDD PDF) 4+ 2019 data.

The intense diffracted peaks were recorded at 2θ = 18.24°; 29.97°; 35.32°; 36.99°; 42.95°; 53.29°; 56.82°; 62.42°; 71.25°; 74.10°, and 78.93°, which denotes the formation of magnetite as a phase pure with a spinel structure. Using the Debye–Sherrer equation (Equation (1)), we were able to calculate the diameter of the magnetic nanoparticles crystallites, at room temperature (24 °C), according to broadening of the most intense peak (311–35.32°) recorded on the XRD graph (Figure 1). According to the XRD data obtained, the width (FWHM) and position (2θ) of peaks, crystallite size (D), lattice constant (a), and distance between crystal planes (d_hkl_) were calculated for the most intense peak (311) at 35.32° on the 2θ axis, and the results are summarized in Table 1.

It is worth noting the larger specific surface area of Fe_3_O_4_@Carbon nanoparticles (S_BET_ = 62 m^2^/g) with an equivalent diameter of D_BET_ = 19 nm. This result is in agreement with the smaller crystallite size of the obtained nanoparticles, calculated using the Debye–Sherrer equation (Equation (1))—D = 20 nm (Table 1).

### 3.2. Raman Spectroscopy

**Figure 2:**
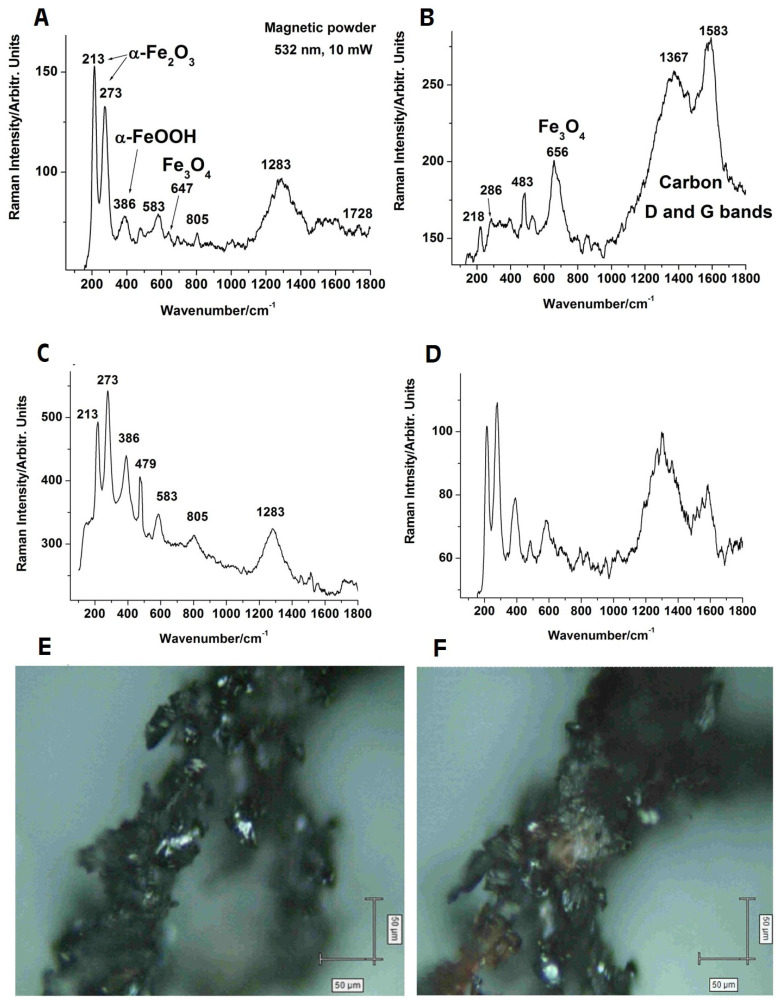
Typical Raman signal collected from different regions of the Fe_3_O_4_@Carbon nanoparticle powder samples (**A**–**D**) under 532 nm excitation (laser spot size ~720 nm); micrographs of the same sample region before (**E**) and after (**F**) laser exposure for spectra collection (20× objective).

Figure 2A–D shows the Raman spectra acquired from different locations on the mag-netic nanoparticles deposited on a glass slide and excited under the 532 nm laser line. Considering that the laser spot size on the sample surface was around 1.8 µm under our acquisition configuration, the spectra indicated an apparent local variation in the sample composition at the micrometer scale. The bulk magnetite (Fe_3_O_4_) core was revealed by the band around 647–656 cm^−1^ [57], while the amorphous carbon coating was revealed by the carbon D (disordered) and G (graphite) bands [58]. Traces of other iron oxide and hydroxide phases, namely, hematite (α-Fe_2_O_3_) and goethite (α-FeOOH), were revealed by the bands at 213–218 and 273–286 cm^−1^, and 386 cm^−1^ [59,60]. Magnetite is known for its wide and low intensity bands [57], while hematite with better crystallinity gives much stronger bands, which may shift slightly to higher wavenumbers due local temperature [59]. Thus, the Raman spectra indeed indicated the synthesis of magnetite nanoparticles with amorphous carbon coating. The optical micrographs taken at the same region of the sample before (Figure 2E) and after (Figure 2F) showed that this nanomaterial in dry powder form was sensitive to laser excitation, changing its appearance after Raman measurements.

The near-infrared laser line at 1064 nm used with the FT-Raman instrumentation did not deliver enough energy to excite the vibrations, including the heavy iron atoms. Hence, only the signal of the graphitic layer covering the iron oxide core could be recorded under the FT-Raman setup.

### 3.3. SEM-EDX Analysis

**Figure 3:**
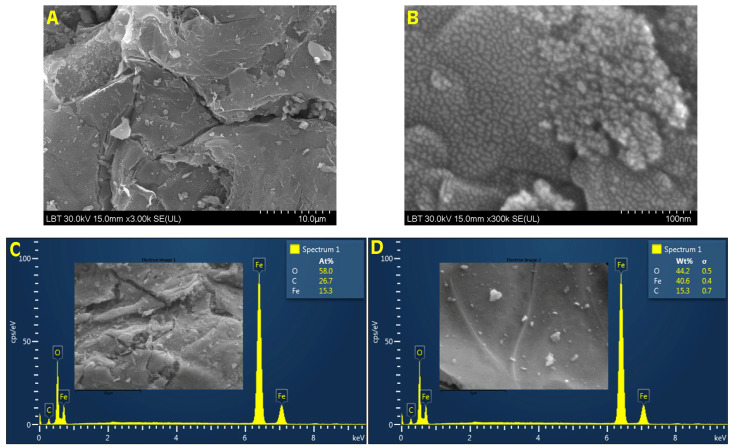
SEM images for Fe_3_O_4_@Carbon nanoparticles synthesized by the combustion method at different orders of magnitude: (**A**)—10 µm; (**B**)—100 nm; (**C**)—EDX spectrum with the atomic percentage of the elements recorded, and (**D**)—EDX spectrum with the weight percentage of the elements recorded.

Figure 3 shows the representative images of Fe_3_O_4_@Carbon nanoparticles obtained at different orders of magnitude (10 µm—to highlight the general aspect of Fe_3_O_4_@Carbon nanoparticles and 100 nm—to highlight the surface topography). As can be seen, at higher magnification (300 kx—Figure 3B), the Fe_3_O_4_@Carbon nanoparticles obtained via the combustion method were highly dense in nature, uniformly distributed, with nearly a spherical shape, and size was in the nanometric scale.

The chemical composition of the element was determined by EDX analysis, and according to Figure 3C,D, the Fe_3_O_4_@Carbon nanoparticles contained only Fe, O, and C, expressed both as atomic percentage and weight percentage values for the elements. Based on the EDX spectrum and the weight percentage values, the iron element was recorded in large quantities. The oxygen was in a slightly higher amount than iron, possible due to the copper of the oxidized grid.

### 3.4. DLS Measurements

**Figure 4:**
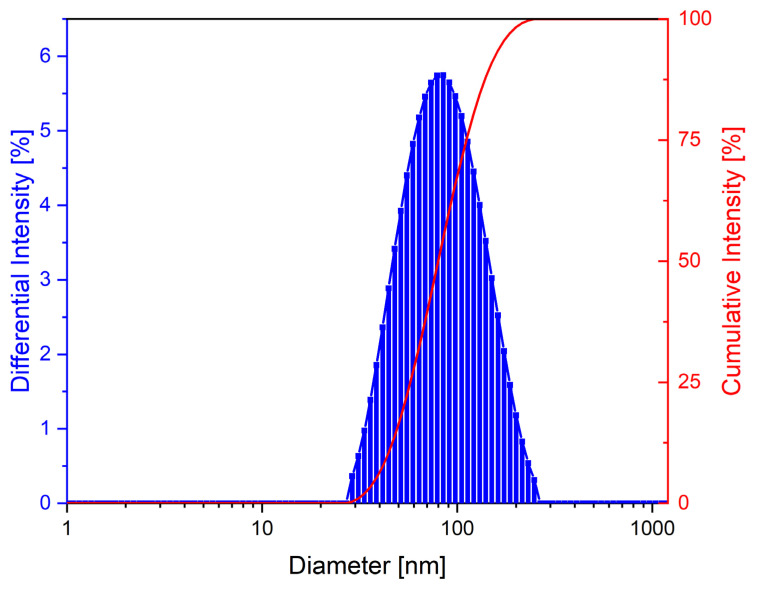
The intensity distribution of the particle size (DLS) of the Fe3O4@C suspension.

In order to assess the particle size and distribution, we prepared an aqueous suspension according to the method described in previous research published recently by our group [61]. The hydrodynamic diameter of the Fe_3_O_4_@C nanoparticles was determined at 25 °C, and from Figure 4 it can be observed that the nanoparticles were monomodal in nature. In addition, the nanoparticle suspension had a narrow size distribution with a mean hydrodynamic diameter of 81.9 nm. The zeta potential obtained (ζ = −47.48) indicated a good stability of Fe_3_O_4_@C nanoparticles in an aqueous medium carrier. Moreover, regarding the heterogeneity of aqueous nanoparticle suspension, the PDI obtained was 0.170. According to the International standard organization (ISO standards ISO 22,412:2017 and ISO 22,412:2017) [62], we can conclude that the Fe_3_O_4_@C nanoparticles were monodispersed in aqueous suspension.

### 3.5. VSM Analysis

**Figure 5:**
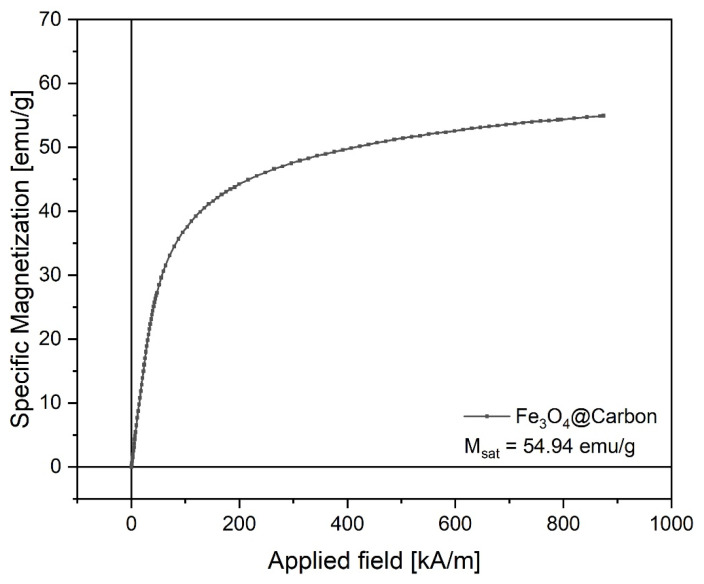
Magnetization curve of Fe_3_O_4_@Carbon nanoparticles.

The VSM data of Fe_3_O_4_@Carbon nanoparticles are shown in Figure 5, and it can be observed that the magnetic nanoparticles synthesized via the combustion method exhibited superparamagnetic behavior at room temperature (24 °C). The saturation magnetization recorded was 54.94 emu/g at maximum field (H_c_ = 900 kA/m). The values recorded for remanent magnetization (M_r_ = 2.7 emu/g) and coercivity (H_c_ = 2.4 kA/m) were very small, which confirms the superparamagnetic behavior.

### 3.6. Biological Profile

In order to evaluate the biosafety profile of Fe_3_O_4_@Carbon nanoparticles, the most used in vitro and in ovo methods were performed, such as (i) cell morphology assessment; (ii) cell viability by means of the Alamar blue assay; (iii) cytotoxicity evaluation by the LDH release method, and (iv) assessment of a possible irritative effect by applying the HET-CAM test. By corroborating the results obtained from the above-mentioned methods, a preliminary biosecurity profile of the Fe_3_O_4_@Carbon nanoparticles can be provided.

#### 3.6.1. Cell Morphology Evaluation

The control cells were represented by the cell monolayer treated with cell culture media and were maintained under the same conditions as the cells exposed to the Fe_3_O_4_@Carbon sample. Thus, the control cells represent the etalon for any morphological alterations that could be induced by Fe_3_O_4_@Carbon nanoparticles.

**Figure 6:**
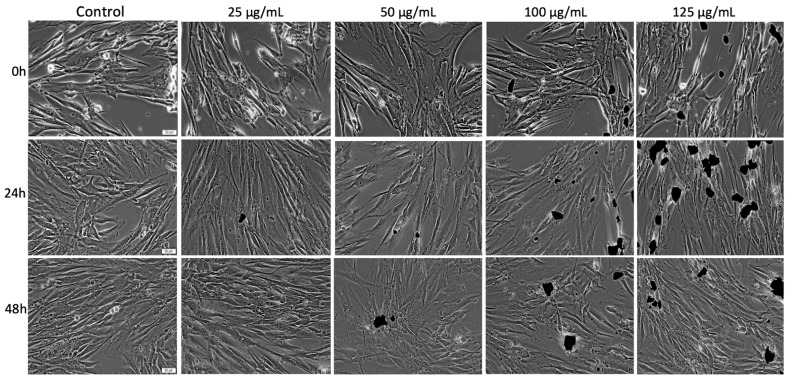
Morphological aspects of the primary human gingival fibroblast (HGF) monolayer—initially (0 h) and at 24 h and 48 h post-exposure to the Fe_3_O_4_@Carbon sample at different concentrations (25 µg/mL, 50 µg/mL, 100 µg/mL, 125 µg/mL). Pictures were taken at a magnification of 20×; the scale bars represent 50 µm. The black particles observed in the images represent the Fe_3_O_4_@Carbon sample.

As presented in Figure 6, the control HGF cells manifested the specific morphological aspects for fibroblasts such as elongated shape with a spindle-like feature and high adherence to the cell culture plate. Regarding the confluence of control cells, it was observed that the confluence increased gradually depending on the incubation time interval, the highest percentage of confluence being recorded after 48 h. Nevertheless, HGF cells treated with the Fe_3_O_4_@Carbon sample at concentrations of 25 µg/mL, 50 µg/mL, 100 µg/mL, 125 µg/mL presented similar morphological aspects as the control cells and no detectable morphological alterations. In addition, the proliferative feature of the HGF cells was not impaired by exposure to Fe_3_O_4_@Carbon nanoparticles, even when the highest concentration of 125 µg/mL was administrated.

**Figure 7:**
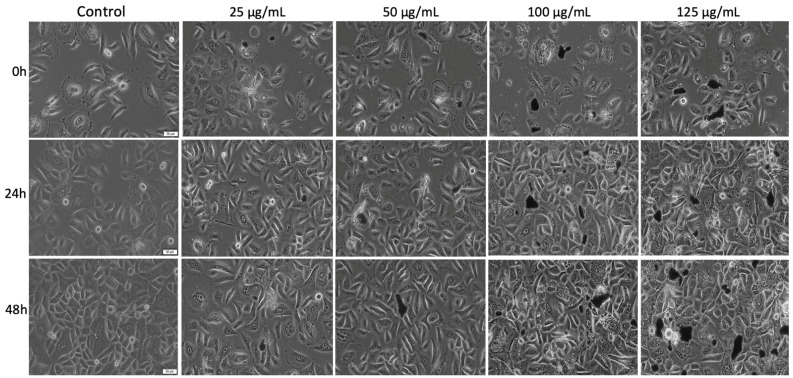
Morphological aspects of primary human gingival keratinocyte (PGK) monolayer—initially (0 h) and at 24 h and 48 h post-exposure to the Fe_3_O_4_@Carbon sample at different concentrations (25, 50, 100, 125 µg/mL). Pictures were taken at a magnification of 20×; the scale bars represent 50 µm. The black particles observed in the images represent the Fe_3_O_4_@Carbon sample.

As shown in Figure 7, the control PGK cells presented an epithelial-like morphology with a cobblestone aspect and rounded features, rather than a flat appearance.

The confluence of PGK cells developed with the incubation time until a cell density of 95–100% was reached at 48 h post-stimulation. However, exposure of the PGK cell line to the Fe_3_O_4_@Carbon nanoparticles at concentrations of 25 µg/mL and 50 µg/mL did not induce significant cell morphological alterations. Nevertheless, when the PGK cell line was treated with concentrations of 100 µg/mL and 125 µg/mL, several cells become rounded and detached from the culture plate (specific signs of apoptosis), especially at 48 h post-stimulation, but the percentage of apoptotic cells was very low.

#### 3.6.2. Cell Viability Assessment

**Figure 8:**
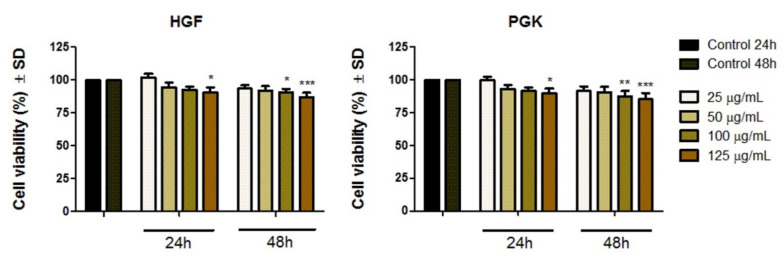
Cell viability percentage of primary human gingival fibroblasts (HGFs) and primary gingival keratinocytes (PGKs) at 24 h and 48 h post-stimulation with the Fe_3_O_4_@Carbon sample at different concentrations (25, 50, 100, 125 µg/mL). The results are presented as mean values of the cell viability percentage (%) normalized to control cells (treated with cell culture media) ± Standard Deviation (SD). One-way ANOVA was applied to determine the statistical differences between test-treated cells and the control, followed by Tukey’s multiple comparisons test (* *p* < 0.05; ** *p* < 0.01; *** *p* < 0.001 *versus* control cells).

The viability percentages (%) of both HGF and PGK cell lines at 24 h and 48 h post-exposure to the 25 µg/mL, 50 µg/mL, 100 µg/mL, and 125 µg/mL Fe_3_O_4_@Carbon samples are presented in Figure 8. As an overview observation, the HGF and PGK cell lines manifested a slight dose-dependent reduction of the cell viability rate; however, the viability of both cell lines did not decrease below 89% after 24 h exposure time. At 48 h post-treatment, the viable HGF population was slightly reduced to a percentage of 86.93%, when 125 µg/mL Fe_3_O_4_@Carbon nanoparticles was applied, while the PGK cell line was more sensitive to Fe_3_O_4_@Carbon nanoparticle treatment, especially when the concentrations of 100 µg/mL and 125 µg/mL were used; in this case the cell viability was 87.47% and 85.2%, respectively.

Nevertheless, no concentration induced an important cell viability decrease of primary human gingival fibroblasts and primary gingival keratinocytes under the current experimental setup.

#### 3.6.3. Cytotoxic Effects by Means of the LDH Assay

In order to provide a more complex biological profile regarding the impact of Fe_3_O_4_@Carbon samples (25 µg/mL, 50 µg/mL, 100 µg/mL, 125 µg/mL) on both cell lines, the LDH release method was also performed to complete the biosecurity portrait of test samples.

**Figure 9:**
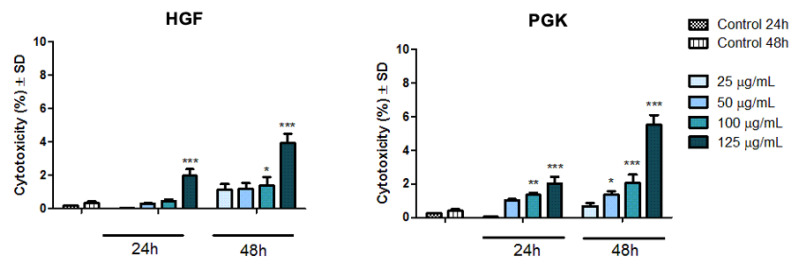
Cytotoxicity rate of primary human gingival fibroblasts (HGFs) and primary gingival keratinocytes (PGKs) at 24 h and 48 h post-treatment with Fe_3_O_4_@Carbon nanoparticles at concentrations of 25, 50, 100, 125 µg/mL. The results represent the mean values ± Standard Deviation (SD) of three individual experiments. One-way ANOVA test was performed to determine statistical differences of sample-treated cells compared to control cells, followed by Tukey’s multiple comparisons analysis (* *p* < 0.05; ** *p* < 0.01; *** *p* < 0.001 *versus* control cells).

As presented in Figure 9, the cytotoxicity rate manifested by both cell lines through lactate dehydrogenase (LDH) quantification into cell culture media followed the same dose-dependent pattern as the one observed for cell viability quantification; the cells presented a slightly increased cytotoxic activity depending on the administrated dose of Fe_3_O_4_@Carbon nanoparticles. For both cell lines—HGF and PGK, the highest percentages of cytotoxic reactions were recorded when the concentration of 125 µg/mL Fe_3_O_4_@Carbon sample was administrated for 48 h; HGF cells manifested a cytotoxic rate of 3.928%, while PGK cells exhibited a cytotoxicity percentage of 5.51%. Nevertheless, this cytotoxic percentage is still extremely low, as compared to the LDH amount released by control cells.

#### 3.6.4. Irritation Potential Using the HET-CAM Assay

Fe_3_O_4_@Carbon nanoparticles at a concentration of 125 µg/mL induced a slight reaction in the vascular plexus of the developing CAM (IS = 4.71) compared to the massive impairment induced by the positive control, SLS (IS = 17.63). 

**Figure 10:**
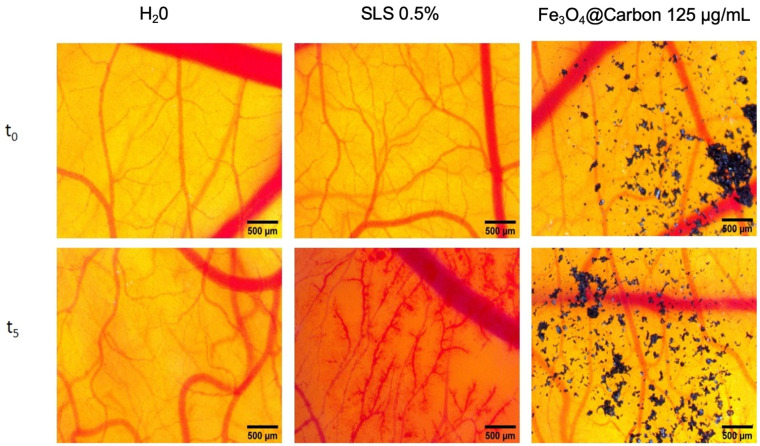
The irritation potential using the HET-CAM method. Stereomicroscope images show the chorioallantoic membrane before (t_0_) and 300 s after application (t_5_) of 300 µL of the test sample of Fe_3_O_4_@Carbon nanoparticles at a concentration of 125 µg/mL and control samples (distilled water as a negative control, SDS 0.5% as a positive control); scale bars represent 500 µm. The black particles observed in the images represent the Fe_3_O_4_@Carbon sample.

According to the irritation scale recommended by Luepke, (0–0.9—non-irritant; 1–4.9—weak irritant; 5–8.9—moderate irritant; 9–21—strong irritant) [56] the Fe_3_O_4_@Carbon nanoparticles can be considered weak irritants at a concentration of 125 µg/mL (Figure 10 and Table 2).

## 4. Discussion

Nanotechnology provides new perspectives in dental medicine to overcome challenging issues caused by complex human diseases and a huge opportunity for the development of new dental products that could be applied in restorative dentistry, implantology, periodontics, edentulism, and even in oral cancers. Of all the nanomaterials suitable for dentistry, magnetic nanoparticles are very seldom studied. In the literature, studies that described the efficient use of magnetic nanoparticles are reported [47,48,63,64], but none refers to the use of magnetic nanoparticles per se. However, Gao and co-workers [65] reported that biocompatible Fe_3_O_4_ nanoparticles exhibited potent anti-biofilm properties without deleterious effects on oral tissues *in vivo*. Based on this preliminary study, two years later, Bukhari and colleagues [63] developed new disinfection technologies based on iron oxide nanoparticles activated with H_2_O_2_ to enhance antibacterial activity on root canal surfaces and in dentinal tubules. Results have shown that iron oxide nanoparticles that were H_2_O_2_ activated were capable of binding to the infected canal surface and inhibited *E. faecalis* presence on canal surfaces and also at different depths of dentinal tubules compared to all other experimental groups. In another research study, Xia and colleagues [64] successfully developed novel iron oxide nanoparticles that were incorporated into calcium phosphate cement scaffolds. They used maghemite and hematite as a source of magnetic material and investigated the effect of the novel calcium phosphate cement scaffolds functionalized with magnetic nanoparticles on human dental pulp stem cell seeding for bone tissue engineering. The results demonstrated that the novel magnetic scaffolds substantially enhanced bone regeneration and osteoinduction, being innovative materials for dental, craniofacial, and orthopedic applications.

Other nanomaterials suitable for dentistry are carbon-based nanoparticles, which are abundant elements with important applications in science and technology fields [66]. Many various carbon allotropes can be synthesized, but the most used classes are fullerenes, nanotubes/nanofibers, and quantum dots. Graphene (an allotrope of carbon) has improved biocompatibility features compared to other classes of carbon-based nanomaterials, but its toxicity has yet not be discussed comprehensively [67]. In addition, the synthesis method involves either mechanical exfoliation/cleavage, chemical vapor deposition or oxidation of carbon precursors in concentrated sulfuric acid [66]. These methods are complicated because they involve other subsequent process, are expensive, and require special or precious materials. Moreover, the quantum dots must be covered with other materials, preventing leaking of the toxic heavy metals, thus allowing dispersion [68].

The current study presents the synthesis of a nanomaterial that combines all the biomedical and physicochemical features of a magnetic material [69] with all the biomedical and mechanical characteristics of graphene [70], more precisely of carbon. In the present research, the combustion method was applied for manufacturing the magnetic nanoparticles based on iron oxides, which have the features required for biomedical applications, namely: biocompatibility, non-toxicity, chemical stability, particle narrow size distribution with a small size resulting in a large surface area, and superparamagnetic behavior [71,72,73,74].

The synthesis of magnetic nanoparticles has received tremendous attention over the years due to the potential applications in nanobiotechnology. For this reason, the employed synthesis method plays an essential role in obtaining the magnetic nanoparticles with appropriate properties for their intended utilization. Mahmudi et al. [75] summarized that the chemical, physical, and biological methods are the three most important published routes for the synthesis of magnetic nanoparticle-based iron oxides. From the chemical route of the magnetic nanoparticle preparation, the most used is the co-precipitation from solution followed by hydrothermal/solvothermal, microemulsion, sonochemical, thermal decomposition, and electrochemical deposition methods [75]. Even if the precipitation method is by far the most simple and used, it has some serious limitations such as the properties of the obtained oxides influenced by many factors such as pH, ionic strength of the solution, temperature, nature of the salts used, the concentration of the precipitating agent or the protective atmosphere. Because of the mutual influence of the working parameters, by using this method, the size distribution of the obtained particles is broad, and their shape and size are hardly controlled [76,77,78,79]. Due to the serious limitations which limit the ability to obtain magnetic nanoparticles with adjustable properties, developing new simple, flexible, and inexpensive methods to synthesize magnetic nanoparticles with tailored properties to provide them with the full potential in biomedical applications is of extreme importance.

A very promising alternative to the methods mentioned above is the solution combustion synthesis that offers several advantages over the other methods. Relying on the exothermic reaction between an oxidizing agent (the desired metal nitrate) and various reducing agents (fuels), this method is simple, energy saving, has a short reaction time, is versatile, eco-friendly, and has a low cost of raw materials and equipment. In the last years, this technique was increasingly used for the preparation of magnetic nanomaterials, especially magnetic iron oxide nanoparticles [43,80,81,82,83]. Magnetite (Fe_3_O_4_) and maghemite (γ-Fe_2_O_3_) are two types of iron oxide nanoparticles with all of the above-mentioned characteristics that are widely used in nanomedicine. Besides the physicochemical features, the magnetic nanoparticles must possess certain biological features, which make them suitable for all areas of nanomedicine, thus overcoming the side effects related to the conventional therapies.

In the current study, various characterization methods have been employed to analyze the physicochemical features of the obtained Fe_3_O_4_@Carbon nanoparticles, namely, Scanning Electron Microscopy (SEM), Dynamic Light Scattering (DLS), X-ray diffraction (XRD), Raman spectroscopy, Vibrating Sample Magnetometry, and Brunauer–Emmett–Teller (BET) techniques.

Following the results obtained after the characterization of the pre-formed magnetic nanoparticles in terms of phase composition, it has been demonstrated that the Fe_3_O_4_ nanoparticles have crystallites size of 20 nm, calculated using the Debye–Scherrer equation (Equation (1)), and magnetite (Fe_3_O_4_) was formed as phase pure without any other secondary phases. The crystallite size is suitable for biomedical application considering the fact that sizes below 100 nm possess lower sedimentation rates, improving tissular diffusion [84]. According to Figure 1, the Fe_3_O_4_@Carbon sample recorded 11 peaks on the XRD pattern at a 2θ scale, representing the corresponding indices of (111), (220), (311), (222), (400), (422), (511), (440), (620), (533), (444) [85]. Our results are in agreement with those obtained by the BET technique, meaning that the equivalent diameter that resulted from the BET method (19 nm) is approximately equal to the diameter of the crystallites (20 nm) after applying the Scherrer equation (Equation (1)). Such results were also reported in the literature, obtained by [47,86]. The phase composition and specific surface area are two parameters that are extremely important because the obtained Fe_3_O_4_@Carbon nanoparticles can be functionalized by combining their surface with biomolecules, materials or drugs, thus enhanced mechanical properties and improving osteogenic potential in vitro and *in vivo*.

The structural analysis by Raman spectroscopy shows the nanomaterial consisting of an iron oxide (magnetite) core with the amorphous carbon coating that was obtained. The broader Raman band at 647–656 cm^−1^ is characteristic of Fe-O vibration within the tetrahedral sites of magnetite [57], while the larger carbon D and G bands testify the presence of abundant carbon material [58]. Hematite and goethite exhibit a higher scattering cross-section, and hence stronger Raman bands, than magnetite due to their greater crystallinity [59], even if they are present in trace concentrations. The lack of XRD peaks of hematite and goethite suggests that these phases are present in quantities below the instrumental detection limit and thus may only be introduced during Raman analysis due to the laser heat input [59].

The medical applications of magnetic nanoparticles also require other parameters which must be controlled in regard to some specific properties. Besides their composition, small crystallites size, and large surface area, the size, morphology, and ultrastructure of nanoparticles have to be even more carefully controlled. Alongside the preparation method employed, the reagent concentration, temperature of the reaction, and stagnation time are of the most critical factors in controlling the size, crystallinity, and shape of the obtained nanoparticles [69]. In this study, nanosized Fe_3_O_4_@Carbon particles with a nearly spherical shape, highly dense in nature, and uniformly distributed (Figure 3B) were obtained. The EDX analysis confirmed the presence of Fe, O, and C in the sample (Figure 3C,D). Nanoparticle stability, an important feature for biomedical applications, was investigated in the current work by measuring the Zeta potential using the DLS technique. According to literature data [87], the closer the Zeta potential is to 0, the more unstable the nanoparticle suspension. The Fe_3_O_4_@C aqueous suspension was highly negatively charged (−47.48 mV) with a narrow size distribution and a single family of nanoparticles. Our results are in agreement with other research studies [61,88,89,90].

Other extremely important characteristics for medical applications, especially for drug delivery, are the magnetic properties and magnetic behavior. Regarding this aspect, Fe_3_O_4_@Carbon nanoparticles with high saturation magnetization were obtained. The negligible values for coercivity and remanence indicate that the Fe_3_O_4_@Carbon nanoparticles obtained exhibited a superparamagnetic behavior. Our findings are in in good agreement with other research studies [91,92,93].

Nevertheless, a preliminary biosecurity profile assessment of the Fe_3_O_4_@Carbon sample, employing a suitable in vitro model, plays an essential role in establishing the clinical potential of these Fe_3_O_4_@Carbon nanoparticles for dental applications.

In vitro models are preferred over in vivo models due to several advantages presented by the cell lines, such us controllable parameters, facile interpretation, reduced expenses, the overcoming of ethical regulations related to animal use for research [52,94].

In the current study, primary human gingival fibroblast and primary gingival keratinocyte cell lines were selected as an in vitro model due to the high percentage of these types of cells in the gingival area and also due to the ability of these cells to simulate reactions observed in in vivo models [95,96]; thus, this in vitro model could provide responses closer to the in vivo oral microenvironment.

Moreover, after reviewing the literature data, no research study has focused on the cytotoxicity evaluation of magnetic nanoparticles with carbon on their surface obtained per se from the synthesis method for relevant cells of oral mucosa, such as primary HGF and PGK cell lines. Thus, the current study provides new relevant data regarding the impact of Fe_3_O_4_@Carbon nanoparticles at the buccal oral mucosa level.

The in vitro results obtained in the present study revealed that the Fe_3_O_4_@Carbon nanoparticles did not induce important cytotoxic effects on the HGF and PGK cell lines under the experimental setup employed (test concentrations of 25, 50, 100, 125 µg/mL and time interval exposure of 24 h and 48 h, where the effect was normalized to control cells). Based on both in vitro colorimetric assays performed (Alamar blue and LDH release methods), the test sample induced a dose-dependent reduction of the viable HGF and PGK cell line populations; the cells exhibiting the most damaging effect when the highest concentration of 125 µg/mL Fe_3_O_4_@Carbon nanoparticles was tested; the HGF cell line manifested a viability rate of 90.46% and a cytotoxic percentage of 1.975%, for a period of 24 h exposure time, while after a contact time of 48 h, the cell viability of HGF cells was 86.93% and the cytotoxic rate was 3.928%. Regarding the biological response manifested by the PGK cell line, according to the current results (Figure 8 and Figure 9), primary gingival keratinocytes seemed to be slightly more sensitive to the Fe_3_O_4_@Carbon nanoparticle treatment, with the PGK cell line exhibiting a cell viability of 89.50% and a cytotoxic rate of 2.027%, when the cells were exposed for 24 h to a concentration of 125 µg/mL Fe_3_O_4_@Carbon nanoparticles and a viability percentage of 85.2%, and a cytotoxicity rate of 5.51% when the same concentration of 125 µg/mL was applied for 48 h. However, by comparing the data resulting from the cell morphology assessment (especially in the case of PGK cell line) with the results obtained from Alamar blue and LDH tests, it can be observed that the cell density developed as the concentration of Fe_3_O_4_@Carbon nanoparticles increased (Figure 7), while the cell viability percentage was slightly reduced when high concentrations of Fe_3_O_4_@Carbon nanoparticles were used (Figure 8), and also, several cytotoxic events were quantified (Figure 9). This phenomenon may be related to the high Fe_3_O_4_@Carbon concentration-treated cells that reached full confluence, in which case the further development of the cell monolayer was compromised, leading inevitably to the cell death of the new cells resulting after full confluence achievement. Thus, this aspect can be translated into a slight cell viability decrease and a quantification of the low cytotoxic rate when compared to control cells, revealing that the toxicological activity noticed was not probably caused by the high concentrations of Fe_3_O_4_@Carbon nanoparticles.

Nevertheless, according to ISO Standard 10993-5:2009 [97], which refers to the biological evaluation of medical devices, one compound could be considered cytotoxic if the viable cell population of the treated cells was reduced by more than 30%. Thereby, the Fe_3_O_4_@Carbon sample could not be labeled as cytotoxic when applied to human gingival fibroblasts and human gingival keratinocytes at concentrations up to 125 µg/mL for an interval of maximum 48 h.

Moreover, the cell morphology assessment of the HGF cell line (Figure 6) revealed that the Fe_3_O_4_@Carbon nanoparticle-treated cells manifested no cytopathic features, as compared to control HGF after 24 h and 48 h post-exposure to all test concentrations (25, 50, 100, 125 µg/mL). Moreover, the proliferative characteristic of the HGF cell line was still active even after the cells were exposed to Fe_3_O_4_@Carbon nanoparticles for a time interval of 48 h; this effect indicates that the test sample was not cytotoxic [98]. However, compared to HGF cells, the PGK cell line exposed to the same treatment conditions (the same concentrations of the Fe_3_O_4_@Carbon nanoparticles—25, 50, 100, 125 µg/mL and identical time exposure—24 h and 48 h) manifested some apoptotic features only when concentrations of 100 and 125 µg/mL were applied for 48 h. Nevertheless, the PGK cell line exhibited no morphological alterations when the cells were treated with these concentrations (100 and 125 µg/mL) for a period of 24 h, or when PGK was exposed to lower concentrations (25 and 50 µg/mL) of Fe_3_O_4_@Carbon nanoparticles for 24 h and 48 h (Figure 7). As already hypothesized above, the cytotoxic events developed by the cells treated with high concentrations of Fe_3_O_4_@Carbon nanoparticles may be caused by the high confluence of the cells that could not further proliferate and may lead to few cell death events that can be quantified as a slight cell viability decrease and a low cytotoxicity rate as compared to control cells. Thus, the cytotoxic aspects recorded may not be related to the impact of Fe_3_O_4_@Carbon nanoparticles on the cell monolayer.

Using the highly vascularized chorioallantoic membrane of the chick embryo, the method involves an evaluation of an inflammatory reaction similar to that induced by a mucosal irritant; therefore, the assay is used for assessing the degree of irritancy of different material ophthalmic preparations, surfactants, cosmetics, dental adhesives, and other various natural compounds and chemicals [50,99,100,101]. The HET-CAM method, an optimal prescreening assay for animal models, represents a simple, low cost and short-term method for predicting the ocular irritant effect of chemicals as an alternative to the classic Draize test in rabbits. It also offers data regarding safety concerns for materials applied to highly vascularized mucosa [102]. Fe_3_O_4_@Carbon nanoparticles showed a slight sign of irritation, localized to a limited area, without influencing the short-term toxicity of the embryo. As shown by our group, a different type of magnetite nanoparticle, a Fe_3_O_4_@OA colloidal suspension, was also found as a moderate irritant in the HET-CAM assay [50], evaluated as a potential permeation enhancement beneficial feature. The Fe_3_O_4_@Carbon nanoparticles could be considered a good biocompatible material. The IS value is close to the moderate irritability category; thus, the concentration of such a material should not be used in a higher concentration for mucosal tissues. The evaluation of nanomaterials by this assay is valuable in terms of estimating the biocompatibility of mucosal materials, offering useful information on the vascular safety of such materials.

Based on these results, the biocompatibility feature of these nanoparticles makes them suitable candidates for various dental applications, such us (i) co-coating material for dental implants providing osteogenic potential when a magnetic field is applied [47]; (ii) co-materials for scaffold-type structures to promote odontogenesis through dental pulp stem cells [64,103]; and (iii) Fe_3_O_4_-hydroxyapatite composites to induce hyperthermic conditions in order to provide a novel approach for malignant bone tumor treatment [104].

## 5. Conclusions

Herein, we proposed the manufacturing of a nanomaterial, obtained in a single step process, in several minutes, at a low cost, with potential use in dentistry, more precisely in regenerative dentistry and bone tissue engineering, drug delivery, and oral cancer treatments. The nanomaterial obtained has both the biomedical features of magnetite and carbon due to the synthesis method employed—the combustion method. The Fe_3_O_4_@Carbon nanomaterial has high specific surface area, suitable for attaching biomolecules or drugs, with a nanoparticle narrow size and a high saturation magnetization, which makes it suitable for targetable drug delivery in oral cancers. Regarding the biosecurity profile, Fe_3_O_4_@Carbon nanoparticles induced no significant cytotoxic effect on human gingival fibroblast and human gingival keratinocyte populations. Regarding the evaluation of the inflammatory reaction, it was found that the Fe_3_O_4_@Carbon nanoparticles induced a slight sign of irritation, localized to a limited area, without influencing the short-term toxicity of the embryo. Based on the in vitro and in ovo methods performed, a preliminary biosafety profile of the Fe_3_O_4_@Carbon was acquired within the limitations of the experimental setup employed.

## Figures and Tables

**Table 1:** Structural parameters of XRD analysis.

	2θ [deg.]	FWHM [deg.]	D [nm]	d_hkl_ [Å]	a [Å]
Fe_3_O_4_@Carbon nanoparticles	35.32	0.283	20	2.5391	8.3200

**Table 2:** Irritation score and type of effect induced by Fe_3_O_4_@Carbon nanoparticles at a concentration of 125 µg/mL.

Test Compound and Controls	Irritation Score (IS)	Irritation Category
Distillate water (negative control)	0	Non irritant
SLS 0.5% (positive control)	17.63	Strong irritant
Fe_3_O_4_@Carbon nanoparticles 125 µg/mL	4.71	Weak irritant

## Data Availability

Not applicable.
